# ACL graft failure location differs between allografts and autografts

**DOI:** 10.1186/1758-2555-4-22

**Published:** 2012-06-14

**Authors:** Robert A Magnussen, Dean C Taylor, Alison P Toth, William E Garrett

**Affiliations:** 1 Department of Orthopaedic Surgery, Sports Health and Performance Institute, The Ohio State University Medical Center, Columbus, OH, 43221, USA; 2 Duke Sports Medicine, Duke University Medical Center, Durham, NC, 27710, USA

**Keywords:** Anterior cruciate ligament, Failure, Location, Autograft, Allograft

## Abstract

**Background:**

Between 5 and 20% of patients undergoing ACL reconstruction fail and require revision. Animal studies have demonstrated slower incorporation of allograft tissue, which may affect the mechanism of graft failure. The purpose of this study is to determine the location of traumatic graft failure following ACL reconstruction and investigate differences in failure patterns between autografts and allografts.

**Methods:**

The medical records of 34 consecutive patients at our center undergoing revision ACL reconstruction following a documented traumatic re-injury were reviewed. Graft utilized in the primary reconstruction, time from initial reconstruction to re-injury, activity at re-injury, time to revision reconstruction, and location of ACL graft tear were recorded.

**Results:**

Median patient age at primary ACL reconstruction was 18.5 years (range, 13–39 years). The primary reconstructions included 20 autografts (13 hamstrings, 6 patellar tendons, 1 iliotibial band), 12 allografts (5 patellar tendon, 5 tibialis anterior tendons, 2 achilles tendons), and 2 unknown. The median time from primary reconstruction to re-injury was 1.2 years (range, 0.4 – 17.6 years). The median time from re-injury to revision reconstruction was 10.4 weeks (range, 1 to 241 weeks). Failure location could be determined in 30 patients. In the autograft group 14 of 19 grafts failed near their femoral attachment, while in the allograft group 2 of 11 grafts failed near their femoral attachment (p < 0.02).

**Conclusions:**

When ACL autografts fail traumatically, they frequently fail near their femoral origin, while allograft reconstructions that fail are more likely to fail in other locations or stretch.

**Level of evidence:**

Level III - Retrospective cohort study

## Background

The anterior cruciate ligament (ACL) is commonly injured and is the most frequently reconstructed ligament of the knee. Reconstructive techniques have evolved over time with variable results [1]. Modern intra-articular reconstructive techniques allow clinically stable ligament reconstruction in the majority of cases; however, failed reconstruction continues to be a problem.

Failure rates of ACL reconstruction are difficult to assess because the definition of failure is not absolute, but many clinical outcome studies place the failure rate between 5 and 20% [1-5]. Although failure of ACL reconstruction is not limited to cases of persistent or recurrent instability [3,6,7], instability is the most frequent reason for revision ACL reconstruction. Johnson et al. classified the etiology of post-operative instability as technical error, failure of graft incorporation, or recurrent trauma [8]. Traumatic re-injury has been reported in 22-28% of patients in several large series [6,9,10]. Traumatic re-injuries may be more common in younger, more active patient populations.

Increased activity level and the use of allograft tissue in ACL reconstruction have been associated with increased graft failure rates [11]. Animal studies have demonstrated slower incorporation of allograft tissue and demonstrated decreased failure loads for allografts up to one year following reconstruction [12]. An understanding of how grafts fail is critical in assessing the reasons for these failures and ultimately in decreasing failure rates. However, we are unable to locate any reports in the literature detailing the location of failure of ACL grafts in the case of traumatic re-injury. We hypothesize that autograft ACL reconstructions will fail near their femoral origin, similar to native ligament tear locations, while allograft reconstructions will fail in other locations.

## Materials and methods

### Patient selection and data extraction

Between February 28, 2006, and March 25, 2010, the two senior authors performed 370 ACL reconstructions, including 44 revision cases. Of these 44 patients, 10 reported no re-injury to their knee prior to the revision ACL reconstruction. These 10 patients were excluded, leaving 34 patients for this analysis. After obtaining approval from our institutional review board, the medical records of these patients were reviewed to verify the diagnosis. Patient demographics, surgical details of the primary ACL reconstruction, time from reconstruction to re-injury, activity at re-injury, time from re-injury to revision reconstruction, and location of graft tear were collected from the medical record. Location of the graft tear was determined from both the operative report and from intra-operative photographs.

### Statistical analysis

Because patient ages, times from primary reconstruction to re-injury, and times from re-injury to revision reconstruction were not normally distributed, they were described in terms of median and inter-quartile (IQ) range rather than as means with standard deviations. Fisher’s exact test was utilized to compare nominal variables between the autograft and allograft groups and a Mann–Whitney *U* test was used to compare continuous variables between the two groups.

## Results

The median age of the 34 patients at primary ACL reconstruction was 18.5 years (Inter-quartile range 16.3 - 22.0 years, overall range 13–39 years). The primary reconstructions included 20 autografts (13 hamstrings, 6 patellar tendons, 1 iliotibial band), 12 allografts (5 patellar tendon, 5 tibialis anterior tendons, 2 Achilles tendons), and 2 unknown. The median time from primary reconstruction to re-injury was 1.2 years (IQ range 0.5 – 3.2 years, overall range 0.4 – 17.6 years). Twenty-seven patients were re-injured during athletic activities, two during military service, and five during other activities. The median time from re-injury to revision reconstruction was 10.4 weeks (IQ range 5.1 – 29.0 weeks, overall range 1 to 241 weeks). No significant differences in these variables were noted between the autograft and allograft groups (Table 1).

**Table 1:** Comparison autograft and allograft patient characteristics

	**Autograft (n = 20)**	**Allograft (n = 12)**	**Significance**
Median age at primary ACL reconstruction (IQ range)	18 years (16 – 21 years)	20.5 years (16.5 – 24.8 years)	p = 0.32
Gender			p = 1.0
* Male*	6 (50%)	11 (55%)	
* Female*	6 (50%)	9 (45%)	
Median time from initial reconstruction to re-injury (years)	1.2 years (0.5 – 2.9 years)	0.9 years (0.5 – 3.2 years)	p = 0.58
Mechanism of Re-injury			p = 0.29
* Basketball*	4	4	
* Soccer*	3	3	
* Other sports*	6	5	
* Non-athletic*	5	0	
* Military Training*	2	0	
Median Time from re-injury to revision reconstruction (weeks)	13.4 weeks (7.0 – 28.1 weeks)	8.4 weeks (2.8 – 35.8 weeks)	p = 0.44
Median age at revision ACL reconstruction (IQ range)	20 years (17.0 – 25.3 years)	22 years (17.8 – 29.3 years)	p = 0.78

IQ = Inter-quartile.

**Figure 1:**
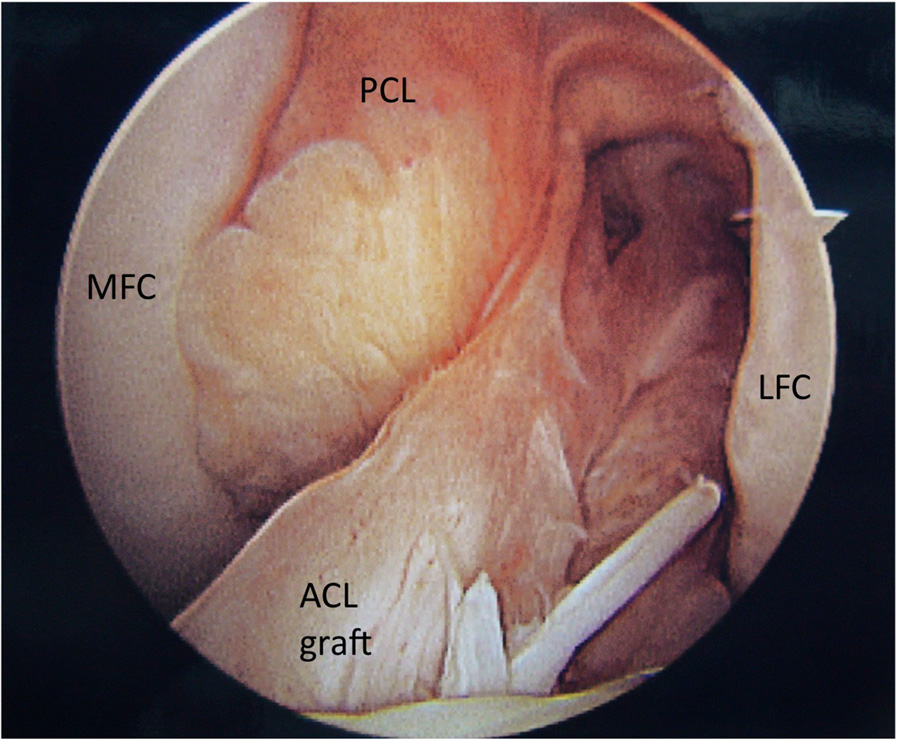
**An arthroscopic view of the femoral notch in a left knee demonstrating rupture of an anterior cruciate ligament (ACL) graft near its femoral attachment.** The medial (MFC) and lateral (LFC) femoral condyles are labeled as is the synovium-covered posterior cruciate ligament (PCL).

**Figure 2:**
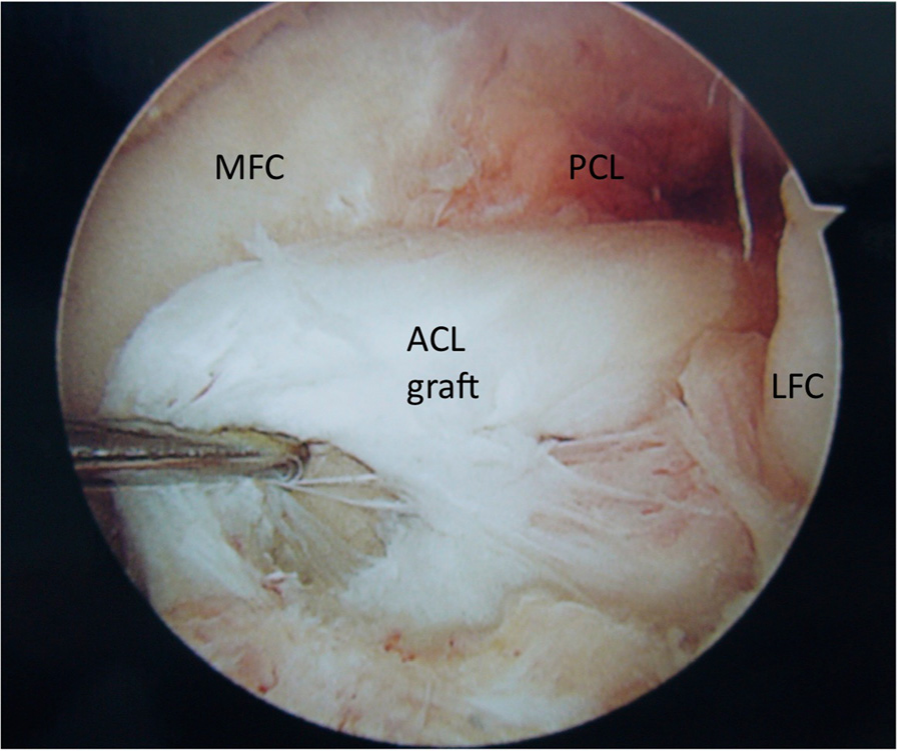
**An arthroscopic view of the femoral notch in a left knee demonstrating rupture of an anterior cruciate ligament (ACL) graft near its tibial attachment.** The medial (MFC) and lateral (LFC) femoral condyles are labeled as is the synovium-covered posterior cruciate ligament (PCL).

**Figure 3:**
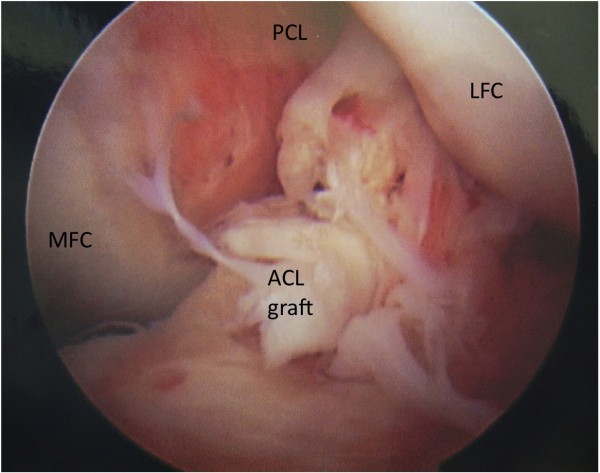
**An arthroscopic view of the femoral notch in a left knee demonstrating rupture of an anterior cruciate ligament (ACL) graft in its mid-substance.** The medial (MFC) and lateral (LFC) femoral condyles are labeled as is the synovium-covered posterior cruciate ligament (PCL).

**Figure 4:**
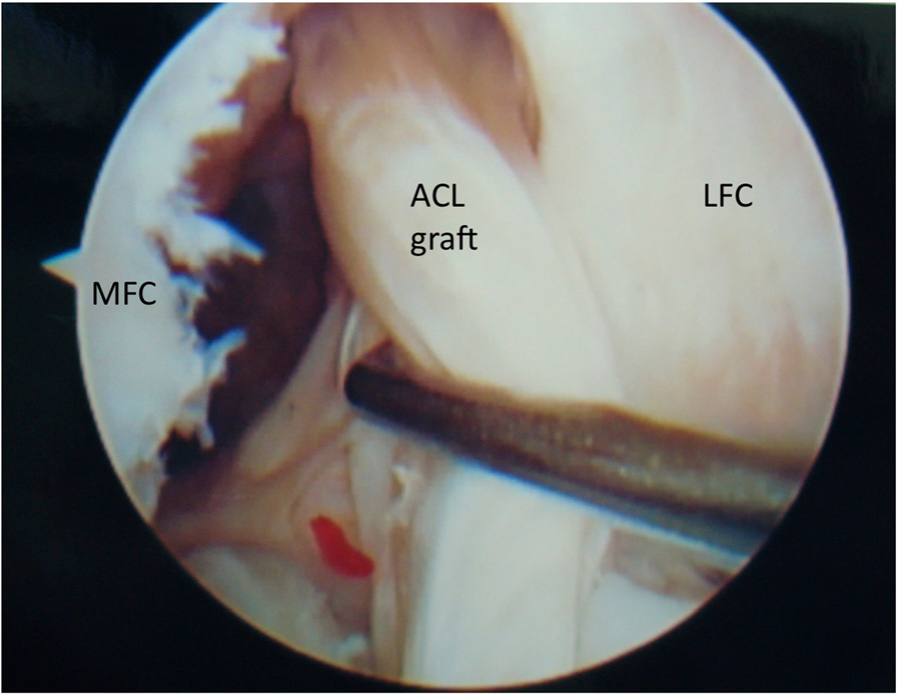
**An arthroscopic view of the femoral notch in a left knee demonstrating stretching of an anterior cruciate ligament (ACL) graft in its mid-substance.** The medial (MFC) and lateral (LFC) femoral condyles are labeled.

**Figure 5:**
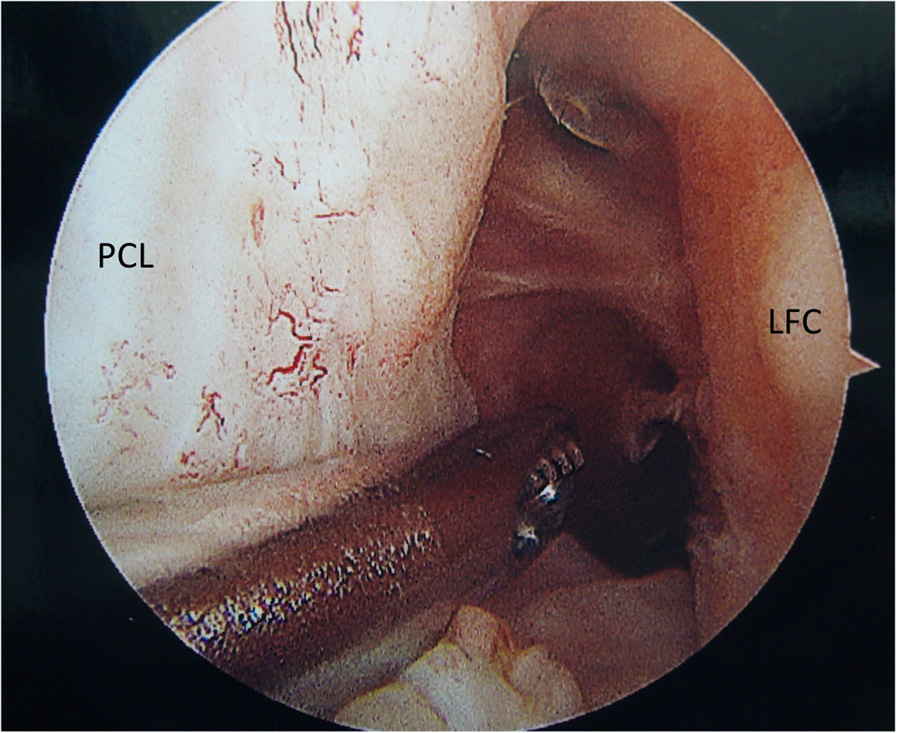
**An arthroscopic view of the femoral notch in a left knee demonstrating disappearance of an ACL graft.** The posterior cruciate ligament (PCL) and lateral femoral condyle (LFC) are labeled.

Graft tear location could be determined in 32 of the 34 patients. Graft tear location was determined by operative report alone in 7 patients, by intra-operative photo alone in 4 patients, and confirmed by both methods in 21 patients. Graft failure near the femoral attachment was noted in 17 patients (Figure 1). Graft failure near the tibial attachment was noted in 3 patients (Figure 2). Graft failure in the mid-substance was noted in 6 patients (Figure 3). A stretched graft without disruption was noted in 4 patients (Figure 4). No graft tissue was seen in 2 patients (Figure 5). We noted no cases in which the graft pulled out of either the femoral or tibial tunnels.

**Figure 6:**
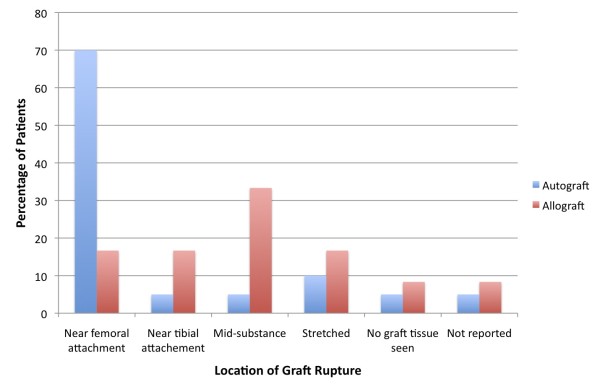
**The distribution of autograft and allograft failure locations following anterior cruciate ligament (ACL) reconstruction.** Autografts are noted to fail most frequently near their femoral origins.

Both primary reconstruction graft type and location of graft failure were known in 30 patients. In the autograft group 14 of 19 grafts failed near their femoral attachment, while in the allograft group 2 of 11 grafts failed near their femoral attachment (p < 0.02) (Table 2, Figure 6).

**Table 2:** Graft failure location by graft type

**Tear location**	**Autograft**	**Allograft**
Near femoral attachment	14	2
Near tibial attachment	1	2
Mid-substance	1	4
Stretched	2	2
No graft seen	1	1

## Discussion

Very little data exist regarding the location of ACL graft failure following traumatic re-injury. It has long been noted that the native ACL, especially the larger anteromedial bundle, frequently tears near its femoral origin, leading to the appearance of the empty wall sign on arthroscopic evaluation [13,14]. One could hypothesize that the propensity the native ACL to tear in this proximal location is related to impingement of the ligament on the roof or wall of the intercondylar notch. The well documented inverse correlation between notch width and risk of ACL tear lends support to this view [15-17], although this correlation could also be explained by the fact having a narrower notch is associated with having a smaller ACL [18,19].

Regardless of the of etiology of proximal tearing in native ligaments, it is likely that a well positioned and ligamentized ACL graft would be predisposed to tear in the same proximal location. The fact that autografts in our series failed in other locations much more frequently than allografts may be explained by slower incorporation of allograft tissue.

The process of revascularization and ligamentization is complex but is felt to follow a regular pattern [20]. Vascular invasion of the graft is noted by three weeks, but the central portion remains poorly vascularized compared to the distal and proximal ends [21]. By eight weeks post-operative, the entire graft is generally revascularized when autograft is used [22]. Numerous animal studies [12,23,24] and as well magnetic resonance imaging (MRI) studies [25] in humans have demonstrated slower revascularization and ligamentization in allografts. Complete healing may never occur in some cases [26]. The increased incidence of graft failure in other locations in allografts in this study strengthens the argument that the increased failure rate of allograft reconstructions may be related to the slower and possibly incomplete ligamentization process.

There are some limitations to this study. Primarily, the retrospective nature of the study limits both the information available regarding the primary ACL reconstruction (including sterilization technique used for allografts, the surgical technique, and rehabilitation protocol used) as well as the information available at the time of revision reconstruction. A prospective study including a careful analysis of graft failure location as well as histologic analysis of remaining graft tissue would be useful in confirming the correlation noted in this study. Additionally, we lacked sufficient data to accurately characterize tunnel locations of the failed ACL reconstructions. It is thus impossible to be certain which patients failed due to a combination of tunnel malposition and traumatic re-injury and which failed due to trauma alone. Further, we identified patients with traumatic re-injury based solely on patient history as document in the medical record. This method may overestimate the number of traumatic graft re-ruptures as patients may attribute re-injury to trivial trauma when then graft was already nonfunctional. The significant delay between re-injury and revision surgery in some cases may have obscured the location of graft failure. Finally, we lack histologic data or other information about the original ACL grafts, limiting our ability to ascertain the reasons for the different failure locations noted in the study.

## Conclusions

When ACL autografts fail traumatically, they frequently fail near their femoral origin, leading to the empty wall sign frequently noted in primary ACL reconstructions Allograft reconstructions that fail are more likely to fail in other locations or stretch.

## Abbreviations

ACL, Anterior Cruciate Ligament; IQ, Inter-quartile; MRI, Magnetic Resonance Imaging.

## Competing interests

Financial competing interests

· In the past five years have you received reimbursements, fees, funding, or salary from an organization that may in any way gain or lose financially from the publication of this manuscript, either now or in the future? Is such an organization financing this manuscript (including the article-processing charge)? If so, please specify. No

· Do you hold any stocks or shares in an organization that may in any way gain or lose financially from the publication of this manuscript, either now or in the future? If so, please specify. No

· Do you hold or are you currently applying for any patents relating to the content of the manuscript? Have you received reimbursements, fees, funding, or salary from an organization that holds or has applied for patents relating to the content of the manuscript? If so, please specify. No

· Do you have any other financial competing interests? If so, please specify. No

Non-financial competing interests

· Are there any non-financial competing interests (political, personal, religious, ideological, academic, intellectual, commercial or any other) to declare in relation to this manuscript? If so, please specify. No

## Authors’ contributions

RAM conceived the study, collected the data, performed the statistical analysis, and wrote the initial draft f the manuscript. DCT performed surgical procedures and aided in revision of the draft. APT performed surgical procedures and aided in revision of the draft. WEG conceived the study, performed surgical procedures, and aided in revision of the draft. All authors read and approved the final manuscript.
